# Convergent Evolution in Aquatic Tetrapods: Insights from an Exceptional Fossil Mosasaur

**DOI:** 10.1371/journal.pone.0011998

**Published:** 2010-08-09

**Authors:** Johan Lindgren, Michael W. Caldwell, Takuya Konishi, Luis M. Chiappe

**Affiliations:** 1 Department of Earth and Ecosystem Sciences, Lund University, Lund, Sweden; 2 Department of Biological Sciences, University of Alberta, Edmonton, Alberta, Canada; 3 Department of Earth and Atmospheric Sciences, University of Alberta, Edmonton, Alberta, Canada; 4 The Dinosaur Institute, Natural History Museum of Los Angeles County, Los Angeles, California, United States of America; Raymond M. Alf Museum of Paleontology, United States of America

## Abstract

Mosasaurs (family Mosasauridae) are a diverse group of secondarily aquatic lizards that radiated into marine environments during the Late Cretaceous (98–65 million years ago). For the most part, they have been considered to be simple anguilliform swimmers – i.e., their propulsive force was generated by means of lateral undulations incorporating the greater part of the body – with unremarkable, dorsoventrally narrow tails and long, lizard-like bodies. Convergence with the specialized fusiform body shape and inferred carangiform locomotory style (in which only a portion of the posterior body participates in the thrust-producing flexure) of ichthyosaurs and metriorhynchid crocodyliform reptiles, along with cetaceans, has so far only been recognized in *Plotosaurus*, the most highly derived member of the Mosasauridae. Here we report on an exceptionally complete specimen (LACM 128319) of the moderately derived genus *Platecarpus* that preserves soft tissues and anatomical details (e.g., large portions of integument, a partial body outline, putative skin color markings, a downturned tail, branching bronchial tubes, and probable visceral traces) to an extent that has never been seen previously in any mosasaur. Our study demonstrates that a streamlined body plan and crescent-shaped caudal fin were already well established in *Platecarpus*, a taxon that preceded *Plotosaurus* by 20 million years. These new data expand our understanding of convergent evolution among marine reptiles, and provide insights into their evolution's tempo and mode.

## Introduction

Mosasaur skeletal remains are fairly abundant in marine deposits of Late Cretaceous age, but the direct fossil record of their soft parts is hitherto confined to a small number of specimens preserving patches of skin [1]–[5], sternal cartilage [3], [6], and portions of the respiratory tube (calcified tracheal rings were initially mistakenly identified as a nuchal fringe [3]) [6]. Consequently, we have a limited understanding of how the mosasaur body plan, tail, and internal organs transformed as these reptiles evolved from semiaquatic dwellers to pelagic cruisers.

The middle Coniacian–early Campanian (∼88–80 million years ago) russellosaurine *Platecarpus* is one of the most common mosasaur genera known from the Western Interior Basin of North America, and arguably a model taxon for understanding mosasaur paleobiology [7]. Much of what we know about *Platecarpus*, and mosasaurs in general, is based on material recovered from the Smoky Hill Chalk Member of the Niobrara Chalk Formation in western Kansas, USA. It was in Logan County, in the upper Santonian to lowermost Campanian part of the stratigraphic column [8], that an exceptional mosasaur specimen (LACM 128319; Natural History Museum of Los Angeles County) was discovered by Marion C. Bonner (unfortunately, there is no date listed for when the fossil was collected; however, it was accessioned to the museum in April 1969). Preserving a wide range of soft tissue structures, LACM 128319 is referred to as *Platecarpus tympaniticus* based on the following suite of distinguishing characters: (1) presence of well-developed median dorsal keel on frontal; (2) presence of narrowly spaced anterolateral processes on frontal; (3) lack of posteromedian flanges on frontal; and (4) distal end of suprastapedial process (of quadrate) transversally expanded [9], [10].

## Results

**Figure 1:**
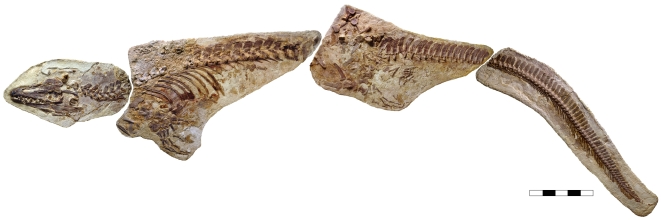
*Platecarpus tympaniticus*, LACM 128319, upper Santonian–lowermost Campanian, Kansas, USA. Specimen photographed under normal light. Scale bar equals 0.5 m.

The largely intact skeleton of LACM 128319, 5.67 m long, has been prepared in oblique left lateral to dorsolateral view (Figure 1). The pelvic girdle and basal part of the tail are somewhat disturbed and some elements in these areas have suffered slight crushing; otherwise, the skeleton is in nearly perfect articulation, preserving the gently curved neck, the broadly hunched back, and the acutely downturned distal half of the tail. Because the skeletal anatomy of *Platecarpus* is reasonably well known [7], [9], the osteology of LACM 128319 will be dealt with elsewhere, and the exceptionally preserved soft tissue morphology and overall body outline are the focus of this report.

**Figure 2:**
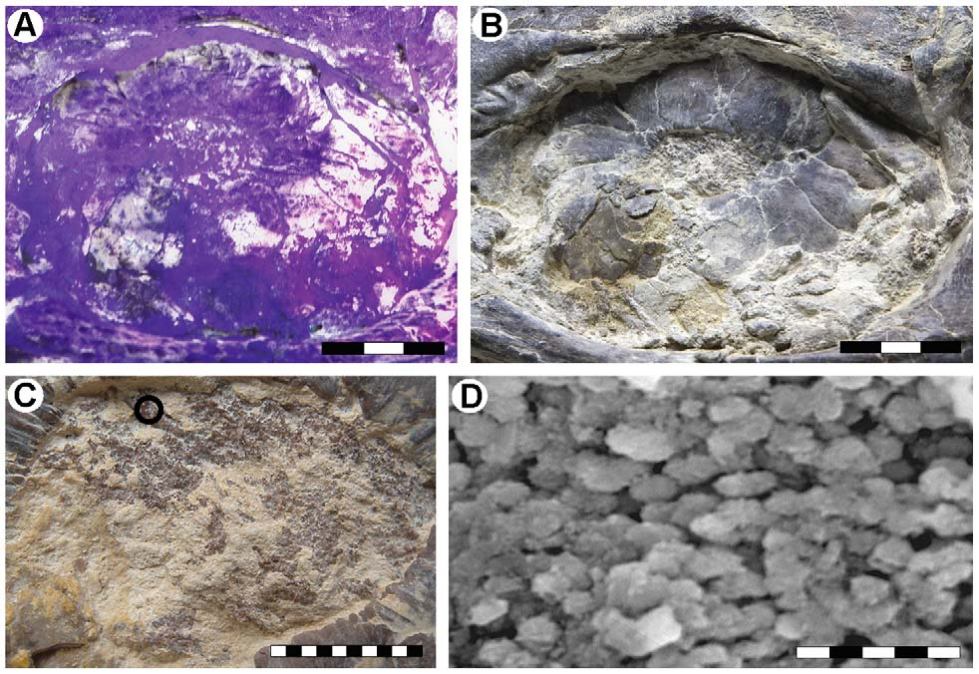
The left eye of LACM 128319. (A) Orbit and scleral ossicles photographed under ultraviolet light. (B) Orbit and scleral ossicles photographed under normal light. (C) Close-up of phosphatized soft tissues (purplish matter, partially obscured by yellow-whitish matrix) pressed against the sclerotic ring aperture, possibly representing remnants of the retina. The area sampled for SEM-EDX analysis is marked with a circle. (D) SEM image of putative melanosomes within the phosphatized soft tissues of the eye. Scale bars represent 3 cm (A, B), 1 cm (C), and 5 µm (D), respectively.

In the head region of LACM 128319, purplish matter in the sclerotic ring aperture of the left eye may represent remnants of the retina, as this tissue was presumably pressed down against the inner surface of the underlying scleral ossicles when the head collapsed during the decay of the carcass (Figure 2A–C). This interpretation is corroborated by the presence of loosely packed, aligned bodies, about 2 µm long, with a morphology that is comparable to that of retinal melanosomes (i.e., lysosome-related organelles of pigment cells) in the eyes of extant tetrapods (Figure 2D) [11], [12]. We refute the possibility that the oblate microstructures represent replacement bacteria because they are embedded inside the fossilized tissues (probably representing their *in situ* position) rather than forming a superficial coating, as would be expected had they instead been part of a microbial biofilm [13]. Moreover, melanosome-like microstructures were not found in any other part of the mosasaur examined under scanning electron microspectroscopy (SEM), including scales, visceral traces, intestinal content, surrounding matrix, and the film that defines the former body outline. Energy dispersive X-ray (EDX) analysis showed that phosphate predominates in the diagenetically mineralized tissue, and hence it is possible that the micrometer-sized bodies are simple microcrystalline apatite aggregates [14]. However, calcium phosphate crystallites often nucleate on already existing soft structures [14], [15], which, in this case, may have been melanosomes. Additionally, apatite aggregates are normally either spherical or cubic in shape [15], not oblate with rounded termini, and, again, would be expected to be found in other phosphatized tissues of LACM 128319 (although we acknowledge the possibility that various types of phosphatization may occur within the same fossil [14]).

**Figure 3:**
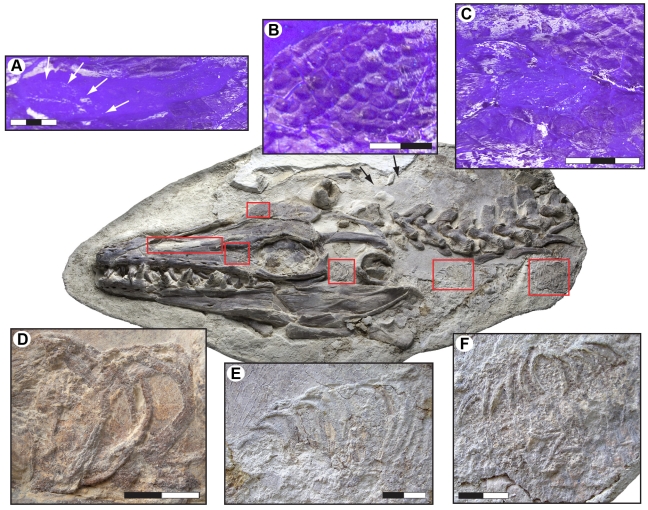
Selected soft tissue structures in the head and neck region of LACM 128319. (A) Close up of the left narial opening under UV light showing skin impressions (scales) covering large parts of the osseous nasal aperture. Arrows indicate the inferred relationship of the fleshy nostril (area without skin covering) to the bony nostril. (B) Scale impressions on the frontal under UV light. (C) Scale impressions on the prefrontal under UV light. (D) Tracheal rings exposed in the left lateral temporal fenestra. (E) Tracheal rings below the cervical vertebral column. (F) Two parallel strings of bronchial rings located below the anteriormost dorsal vertebra. Scale bars equal 3 cm (A, C) and 2 cm (B, D–F), respectively.

Portions of the respiratory tube, as represented by its reinforcing cartilaginous rings, are visible in the temporal region of the skull through the posterior portion of the neck, immediately anterior to the pectoral girdle. Tracheal rings [diameter = 26.2–34.4 mm (taphonomic compression gives widely varying measurements)] first appear along the lower half of the lateral temporal fenestra (Figure 3D). They are concealed under the left quadrate, but reappear between the retroarticular processes of the lower jaws. Following an abrupt upward turn suggesting dislocation prior to burial, another section of the trachea occurs some distance ventral to cervical vertebrae four and five (Figure 3E). Unfortunately, the segment in which the trachea bifurcates into right and left bronchus was lost during quarrying. Nonetheless, two sub-parallel strings of bronchial rings (average diameter about 20 mm) situated below the first dorsal vertebra suggest that mosasaurs, similar to extant limbed squamates, had two functional lungs (Figure 3F). Snakes, on the other hand, only have one functional lung (the left one is either vestigial or absent altogether) because their tubular bodies require their internal organs to be reduced in thickness and/or in number [16]. Apparently, the tracheal bifurcation occurs anterior to the forelimbs in mosasaurs, unlike the condition in terrestrial lizards in which the bifurcation occurs in the chest region at the level of the forelimbs [17]. Among mammals, whales also exhibit a short trachea (due to their abbreviated neck) supported by heavy cartilaginous rings, followed by paired bronchi that in some taxa extend sub-parallel to each other rather than diverging from one another [18], [19].

**Figure 4:**
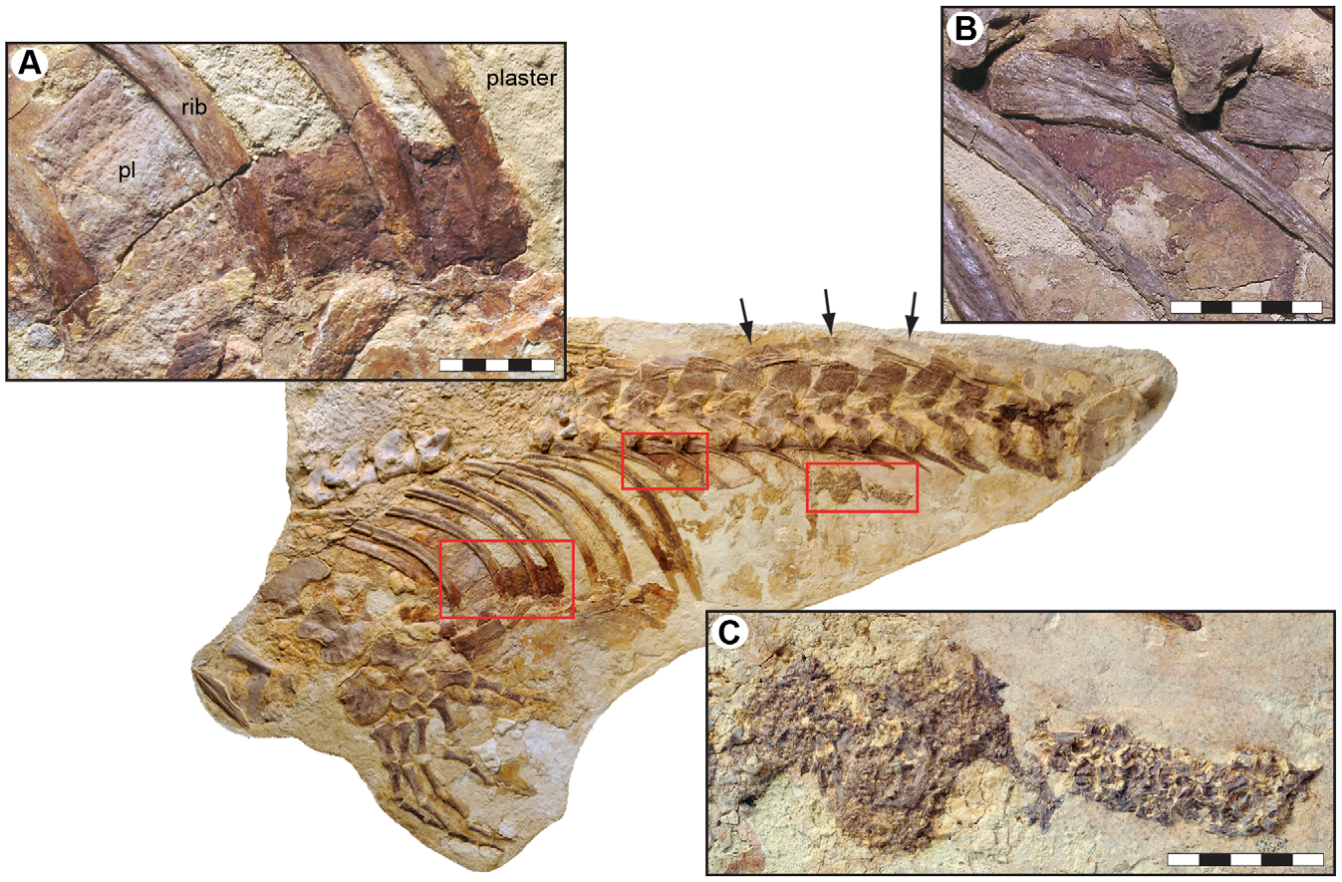
Putative visceral traces in the thoracic and abdominal cavities of LACM 128319 (arrows indicate preserved body margin). (A) A large, reddish pigmentation within the lower rib cage. pl = intercostal plate. (B) Another smaller discoloration located high in the anterior portion of the abdomen. (C) Gastrointestinal content in the form of densely packed and partially digested fish bones. Scale bars represent 5 cm.

In the area of the lower rib cage, a reddish stain extends from the third to fifth thoracic rib (Figure 4A). The colored area is bounded anteriorly by the third rib, which, in turn, is located posterior to a thin sheet of mineralized tissue (possibly the remains of an intercostal plate; ‘pl’ in Figure 4A). Consequently, it is possible that the trace continues forward some distance inside the rock. Ventrally, the pigmentation disappears underneath the calcified cartilage of the sternal ribs to suggest that it was something inside the body cavity that remained within the rib cage as the animal decomposed (thus excluding the possibility that the red matter is a residual of microbial mats that grew on the underside of the body). Its present dorsal margin, however, is an artifact produced by plaster-filling in the space between the long ribs. Likewise, it is currently not possible to confidently determine the original posterior edge of the stained area due to cement infill, although a reddish coloration on the succeeding long ribs would suggest that it may have been at the level of the eighth thoracic rib.

Another ruddy patch is located ventromedial to the second and third lumbar vertebra, lining the medial surface of the left rib head of the second lumbar (Figure 4B). The colored layer is bounded above by the vertebral centra, and is interrupted anteroventrally by the left rib of the first lumbar (presumably an artificial perimeter because there is cement infill in the area immediately anterior to this rib). Ventroposteriorly, the colored matter gradually thins out and fades away; hence, there are no precise edges.

A previous chemical analysis of the aforementioned pigmentations, using mass spectrometry and X-ray diffraction techniques, detected sufficient amounts of iron and porphyrin-derived compounds suggestive of the presence of hemoglobin decomposition products, and thus indicative that the traces may represent residues of visceral organs derived from the decaying animal [20]. The heavily pigmented matter does have a substance and consistency that are noticeably different from those of the surrounding bones and matrix. Our SEM-EDX analysis demonstrated that the stained areas contain iron, oxygen and carbon, to suggest a partial replacement of the organic matter with either siderite or pyrite (which, in turn, may have altered to iron oxyhydroxides [21]), i.e., diagenetic minerals commonly associated with exceptional soft tissue preservation [22]. Decomposition and subsequent compaction during burial may have displaced the organic residues toward the lower part of the body wall, thereby explaining the discoloration of the adjacent ribs (Figure 4A).

High in the abdomen, well in front of the pelvis, lie the contents of the gastrointestinal tract. These consist of partially digested remains of moderate-sized fish packed into a dense mass with an outline that appears to follow the course of the digestive tract (Figure 4C). It is possible that the ingested bones derive from the anterior portion of the digestive system (they would then represent displaced stomach contents); however, given that the longitudinal axis of the well-delimited skeletal accumulation runs dorsally beneath and parallel to the vertebrae in the lumbar region, it is more likely that the residues represent processed food from within the colon. Given this, the incompletely digested bones would suggest that mosasaurs, similar to e.g., tyrannosaurid theropod dinosaurs [23], had short gut-residence times and/or low gastrointestinal absorption rates (which would then also explain the sporadic finds of massed bivalve shell pieces in gastric residues and alleged coprolites of the durophagous mosasaur *Globidens* [24]). Alternatively, the resistant skeletal elements were rapidly transported through the digestive system as waste material poor in nutrients [25].

**Figure 5:**
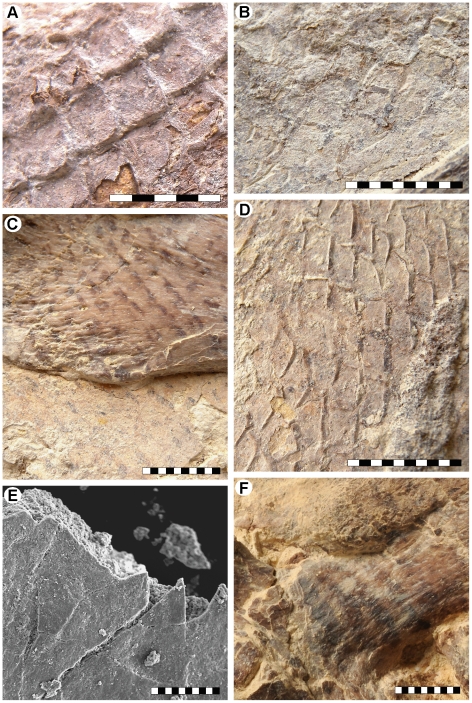
Integumentary structures of LACM 128319. (A) Articulated three-dimensionally preserved scales above the fifth cervical vertebra. (B) Two-dimensionally preserved scales above the third cervical vertebra. (C) Scale impressions on the left rib head of the sixth cervical vertebra. (D) Three-dimensional scale impressions in the gular region. (E) SEM image showing flattened and phosphatized remains of a two-dimensionally preserved scale on top of matrix. (F) Putative color markings on the third cervical vertebra, or, alternatively, differential preservation of the skin impressions. Scale bars equal 5 mm (A), 10 mm (B–D, F), and 100 µm (E), respectively.

**Figure 6:**
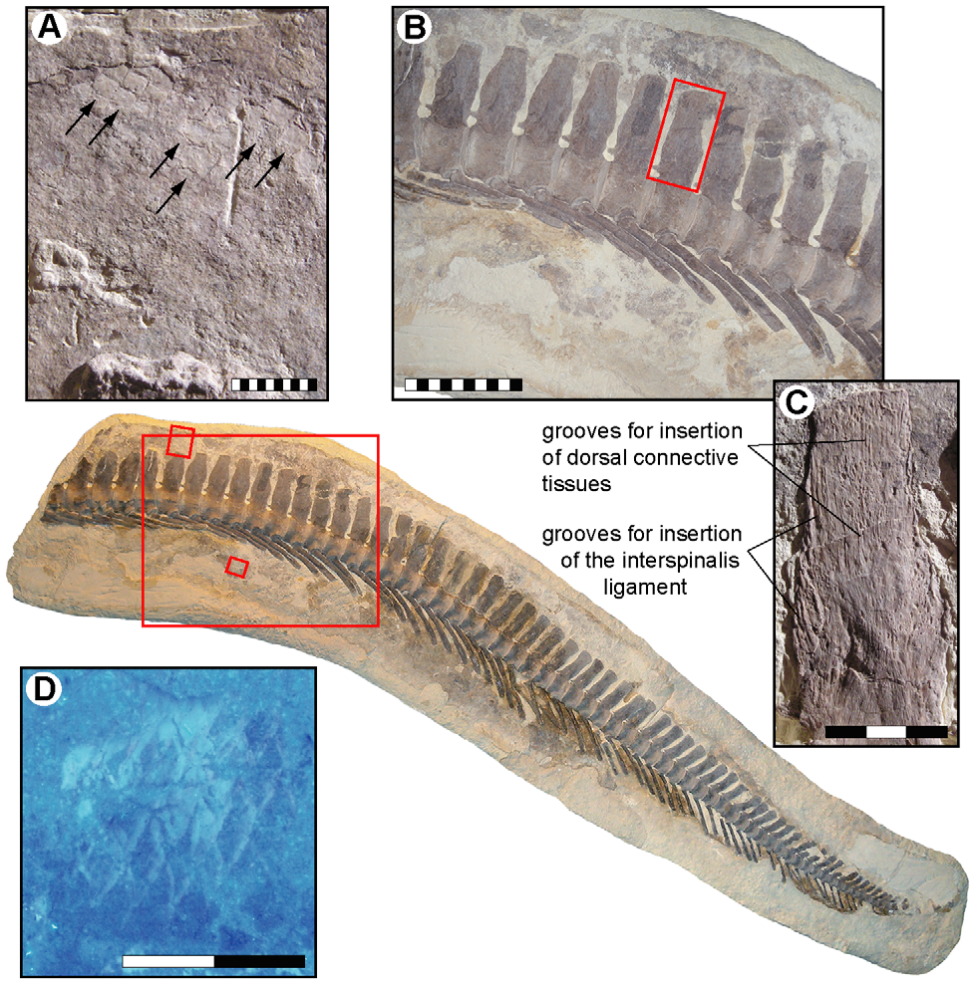
Fluke area of the tail of LACM 128319. (A) Scale impressions (arrows) above the last intermediate caudal vertebra at the base of the caudal fin. (B) Natural tailbend as indicated by the presence of wedge-shaped vertebrae (see also Figure 7A). Note the horizontal inclination of the haemal arch-spine complexes on the left side of the image, signaling the presence of a narrow caudal peduncle, and how these complexes gradually become more ventroposteriorly directed down the tail. Also note transition from procumbent to recumbent neural spine orientation, matching that of the axial support in *Plotosaurus* and, albeit inverted, modern selachians with semilunate caudal fins [28]. It is also evident that the neural spines and vestigial prezygapophyses are evenly spaced throughout the tail when it is bent downwards; consequently, aligning the vertebrae in a horizontal line would require these vertebral processes to overlap with each other. (C) Longitudinal and transverse grooves on a neural spine (inset in B), probably representing insertion points for the interspinalis ligament [7] and connective tissues associated with a dorsal plate that may have contributed to a more streamlined cross-section of the ventral lobe (a similar structure of connective tissue is found in extant aquatic monitor lizards [40]), (D) Columnar scales below the fourth terminal caudal vertebra under UV light. Scale bars represent 1 cm (A), 10 cm (B), 3 cm (C), and 2 cm (D), respectively.

The most remarkable features of LACM 128319 are the preservation of skin structures from all parts of the body and the undistorted posture of the caudal fin. The squamation is preserved as articulated sections of phosphatized matter and faint impressions along the neck, abdomen, and upper and lower surfaces of the tail (Figures 5A, B, D, E, 6A, D), and as a reticulated pigmentation on the bone surfaces (Figures 3A–C, 5C). Some parts of the integument, particularly on the head, are poorly discernable in normal light but fluoresce under ultraviolet light (e.g., Figure 3A–C).

The scales covering the tip of the snout are large (approximately 10 mm when measured transversely), non-imbricated, and sub-hexagonal in outline. Along the upper and lower jaws the scales are longitudinally rhomboidal (measuring up to 20 mm in length), and they are obliquely arrayed into an alternating pattern where neighboring scales overlap one another. Large, rhomboidal scales also cover the posterior, gently domed portion of the left osseous nasal aperture (Figure 3A), to suggest that the fleshy nostrils were situated far anteriorly, as well as somewhat peripherally in mosasaurs, as they are in most extant squamates and archosaurs [26]. The scales in front of the orbit are longitudinally oval in shape (Figure 3C), whereas those bordering this cranial concavity from above and below have more-or-less rhomboidal outlines (Figure 3C). At the top of the skull the scales appear as shallow, hexagonal depressions (Figure 3B). The scales covering the anterior end of the frontal are reasonably large (about 7 mm when measured diagonally); however, they diminish in size posteriorly and gradually attain the dimensions of the body scales.

**Figure 7:**
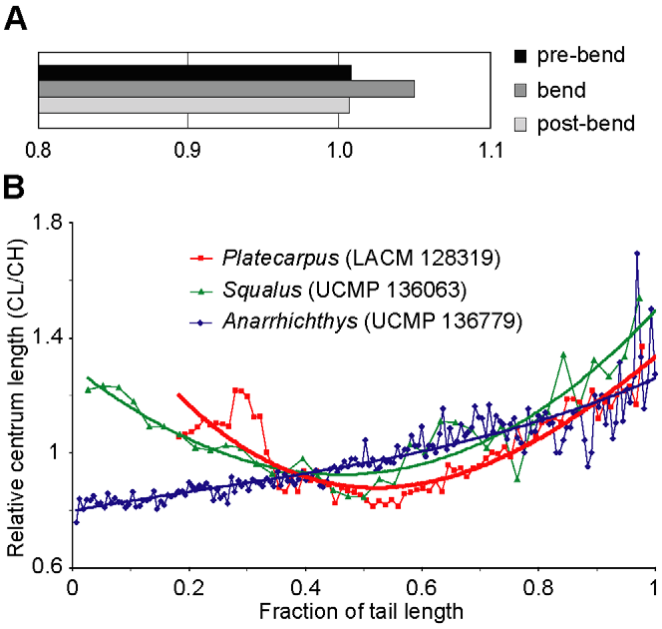
Morphometric data for LACM 128319, an extant shark (*Squalus*), and the wolf eel *Anarrhichthys*. (A) The ratio of dorsal centrum length to ventral centrum length at the tailbend of LACM 128319, in thirteen caudals that precede the bend, and in thirteen vertebrae that succeed it (see Table S1 for measurements). Even though the differences in dorsal versus ventral centrum length are not readily visible in individual vertebrae, the cumulative effect accounts for a considerable ventral flexure given the length of the downturned segment. (B) Relative vertebral centrum length (CL/CH) and change in vertebral dimensions along the caudal segment of the vertebral column of *Platecarpus* (LACM 128319), *Squalus* (UCMP 136063; University of California Museum of Paleontology, Berkeley), and *Anarrhichthys* (UCMP 136779). Polynominal order of the regression curves = 3. R^2^ values = 0.782 (LACM 128319), 0.826 (UCMP 136063), and 0.799 (UCMP 136779), respectively. See main body of text for interpretations.

Whereas the head scales include a blend of morphologies, the body scales are all rhomboidal, well imbricated, and arranged in an alternating pattern where adjacent rows are diagonally offset from one another. As in the advanced mosasaurine *Plotosaurus* [5], the posterior scale margin appears to be acutely medially angled (Figure 5A); yet there are no apparent keels or other external surface ornamentations. The body scales measure about 3.6×3.3 (width×length) to 4.4×4.4 mm along the dorsal surface of the neck, 5.5×5.2 mm above the anterior tracheal rings, 4.4×3.0 to 4.8×3.1 mm along the ventral margin of the rib cage, 4.3×3.9 to 4.7×4.3 mm over the posterior dorsal region, and 4.4×4.6 to 5.5×4.0 mm at the tail base; hence not showing any significant size gradation across the body. The size (up to 10.5×4.7 mm) and morphology (tall, almost columnar) of the caudal scales in the hypaxial region of the tail fin differ somewhat from those of the body scales (Figure 6D), whereas the epaxial caudal scales are comparable in size (about 3.0×3.4 mm) and morphology (regular rhomboidal) to the body scales (Figure 6A). Paddle scales are also comparable in morphology and organization to the body scales but appear to become progressively larger distally. Putative color markings, in the form of darkly pigmented, irregular stains and narrowly spaced, oblique stripes (Figure 5F), are found on the premaxilla, third cervical vertebra, and along the gum line. A dark, phosphatic film defines the fleshy outline of the neck (Figure 3 – black arrows) and right body margin (Figure 4 – arrows). Unfortunately, most skin structures surrounding the tail fin were lost during collecting and/or initial preparation of the specimen before the recognition that soft tissues were preserved. It is noteworthy, however, that scale impressions above the neural spines extend to the edge of the block that contains the posterior tail portion of LACM 128319 (Figure 6A). Additionally, the terminal caudal segment is structurally downturned due to the presence of wedge-shaped vertebrae (i.e., vertebrae where the dorsal centrum edge is longer than the ventral centrum edge; Figures 6B, 7A) [27], [28], preserving the original configuration of the arcuate fringe formed by the neural spines in the anterior terminal caudal series near the bend (Figure 6B).

## Discussion

**Figure 8:**
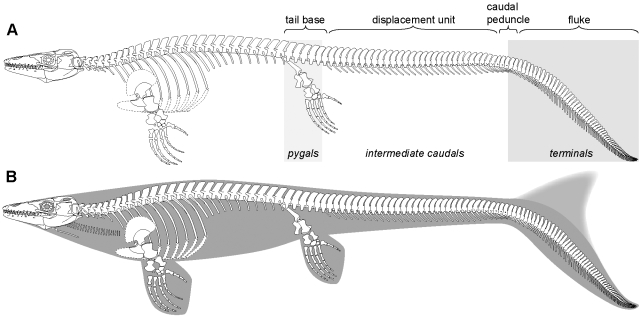
Skeletal reconstruction and inferred body outline of the plioplatecarpine mosasaur *Platecarpus*. (A) New reconstruction based on LACM 128319. Note the regionalized caudal vertebral column resulting in four discrete structural units: proximal tail stock or base, mid-sectional displacement unit, narrow caudal peduncle, and distal fluke or propulsive surface. Also note the change in inclination of the neural and haemal spines along the caudal segment and the distinct tailbend formed by wedge-shaped vertebrae. The caudal terminology follows that of [28]. (B) Inferred body form of *Platecarpus*. The precise shape and depth of the dorsal lobe of the caudal fin is unknown.

The anatomy of the tail of LACM 128319 provides compelling evidence to suggest that the posterior caudal segment of *Platecarpus* was modified to form a hypocercal caudal fluke (i.e., an asymmetric tail fin where the axial support is bent downwards), and provided the main propulsive forces during swimming (Figure 8).

The presence of a deep caudal fin in *Platecarpus* is corroborated by meristic changes in vertebral centrum dimensions along the tail segment of LACM 128319 (Figure 7B). Modern anguilliform swimmers equipped with oar-like tail flukes, such as the wolf eel *Anarrhichthys*, have vertebral centra that increase progressively in length posteriorly to amplify the thrust-producing flexure of the tail (Figure 7B). This is in stark contrast to sharks with epicercal (two-lobed) tail fins, where the relative length of the caudal centra decreases distally to a point a short distance posterior to the greatest depth of the caudal fluke (i.e., at the level of the unsupported ventral lobe). Beyond this point the centra become progressively longer, resulting in a centrum length/centrum height (CL/CH) ratio curve with a pronounced dip at its center (Figure 7B – *Squalus*). In LACM 128319, the relatively shortest centra are situated at the level of the tailbend below the fan-shaped structure formed by the elongate neural spines; a condition that compares well with that of extant sharks (Figure 7B). Presumably, the short, disk-shaped centra contributed to enhanced stability at the crest of the fluke, a portion of the tail that must have been under great stress when the animal was swimming.

As demonstrated by Lindgren et al. [28], the tails of derived mosasaurs can be subdivided into four discrete subregions (stable proximal tail base, mid-sectional displacement unit, narrow caudal peduncle, and distal propulsive surface or fluke) based on regional variations in vertebral centrum dimensions and discrepancies in vertebral process length and spine orientation (Figure 8A). Building on this functional model, and with additional observations from LACM 128319, it is inferred that fluke displacement was accomplished by sideways excursions of the intermediate caudal segment where the neural spines are elongate and consistently posteriorly inclined (Figure 8), and originally were probably interconnected to adjacent neural spines by collagenous fibers (i.e., interspinalis ligaments; Figure 6C) [7]. This arrangement would have acted as a series of springs, storing energy, and returning the tail to the rest position. A similar arrangement is found in modern fish [29], where it presumably conserves energy during rhythmic bending movements. It is further assumed that the preceding tail base (formed by the tallest and widest column centra in the caudal vertebral series, i.e., the pygals; Figure 8A) acted as a reinforced foundation upon which the undulations took place [28]. Moreover, judging from the preserved ventral outline impression and changes in the orientation of the haemal spines (Figure 6B), the distal tail blade was hinged upon a relatively narrow caudal peduncle, and thus offset from the anterior, somewhat deeper portion of the tail (Figure 8).

A hypocercal tail fin also occurs in other derived pelagic reptiles, notably ichthyosaurs [30] and metriorhynchid crocodyliforms [31], and strongly implies a common pattern of adaptation towards an obligate marine existence. In cetaceans, ichthyosaurs, and metriorhynchid crocodyliforms, the stringent hydrodynamic constraints imposed by the surrounding water provided important selections that, with time, optimized their body plans for effective axial undulatory locomotion (i.e., they changed from being anguilliform/sub-carangiform to carangiform, and, in some derived taxa, even thunniform swimmers) [32]–[34]. Accordingly, early members of these groups radically modified their tails, stiffened their backbones, and reduced their rear limbs (though not in crocodyliforms) to meet the demands of marine life [30]–[34].

**Figure 9:**
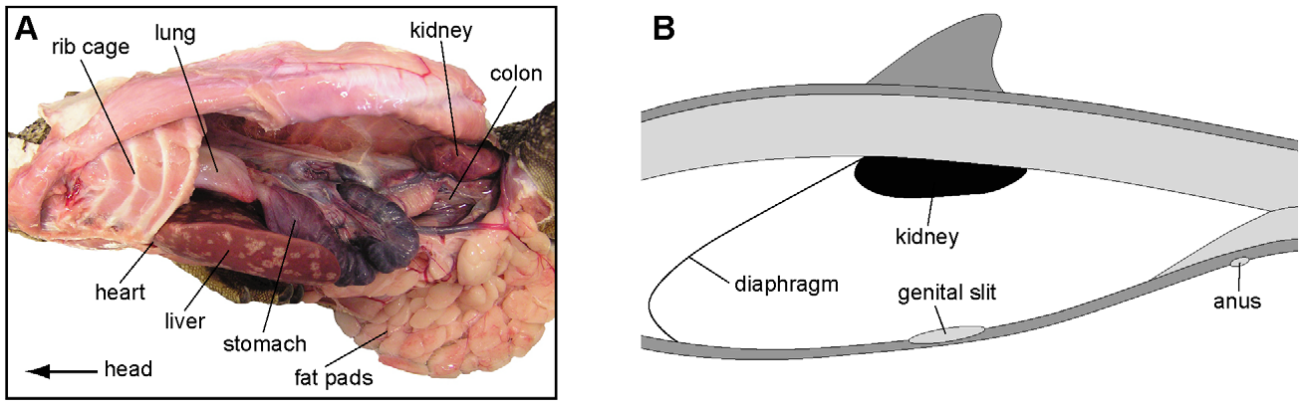
Visceral positions within the thoracic and abdominal cavities of a monitor lizard and a cetacean. (A) Dissection photograph showing the location of the internal organs within the body cavity of the extant monitor *Varanus exanthematicus* (LO 10298). (B) Longitudinal section through the abdomen of a cetacean showing the location of the kidneys (modified from [36]).

When compared to modern terrestrial lizards (Figure 9A), the position of the large reddish stain within the lower rib cage (Figure 4A) correspond well with that of the heart and/or liver, to suggest that it may be a trace of one, or both, of these organs. Additionally, the second pigmentation, i.e., the one located high in the abdomen (Figure 4B), could potentially be the residual of a kidney, as has previously [20] been suggested. This would, however, require an anterior migration of this organ compared to its position in extant monitors (Figure 9A). Interestingly, the kidneys are located immediately behind the diaphragm in cetaceans, near the transition from the thoracic to lumbar vertebral series (Figure 9B) [35], [36]. Thus, the more anterior locations of the inferred kidney and intestinal content in LACM 128319 would indicate a forward migration of the rib cage, a condition common to derived mosasaurs [7]. We suggest that transformation produced a similar body profile in mosasaurs to that of cetaceans [35], [36], in which the abdominal viscera is situated anteriorly in order to contribute to a more streamlined body profile.

We hypothesize that the small-sized and morphologically homogenous body scales of LACM 128319 may have served to stiffen the body and to resist axial compression during rhythmic bending movements, thereby providing a hydrostatic structure that maintained the shape of the animal when it was swimming [29], [37]. Moreover, judging from the preserved squamation in LACM 128319, the primitively-limbed mosasauroid *Vallecillosaurus* [4], and highly piscine mosasaurine *Plotosaurus* [5], there was a gradual reduction in both relative and absolute body scale size with increased marine specializations in mosasaurs, to suggest an adaptation for improved hydrodynamic efficiency by minimizing friction drag when water flowed past the body [29].

**Figure 10:**
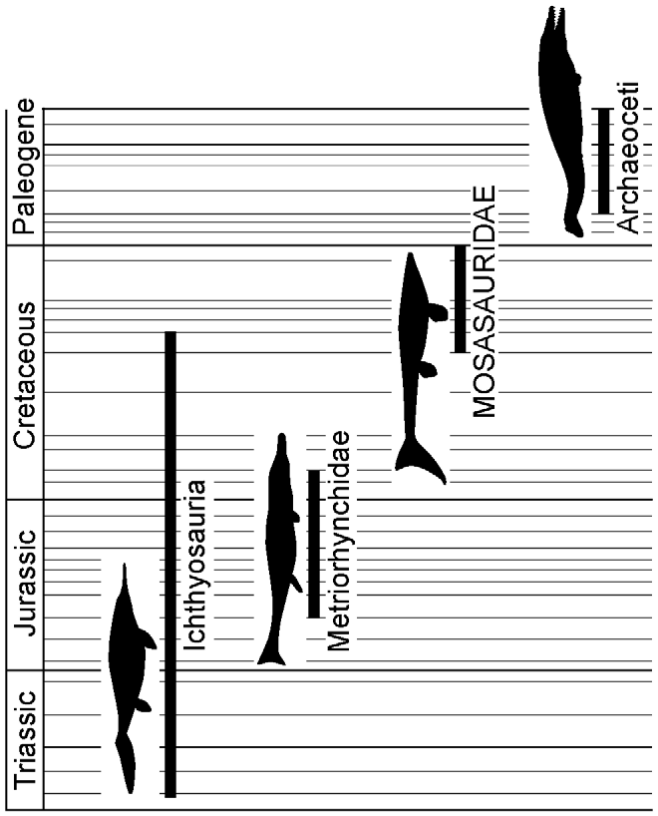
Temporal durations of Ichthyosauria, Metriorhynchidae, Mosasauridae, and Archaeoceti together with silhouettes of a moderately derived form of each clade (not to scale). The body profiles represent forms known from approximately 10 million years after the initial stratigraphic appearance of respective group and are based on LACM 128319 (Mosasauridae) and restorations in [32]–[34]. These forms presumably utilized carangiform locomotion, as opposed to anguilliform and/or sub-carangiform propulsion as did their predecessors [28], [30]–[34].

While a precise tempo of secondary aquatic evolution among reptiles remains elusive, the fossil record clearly shows an exciting and important correlate between mode (convergent evolution) and tempo (time to achieve a fusiform body shape): ichthyosaurs, metriorhynchid crocodyliforms, and mosasaurs (as well as whales among mammals) all achieved their variants on a streamlined body form early in their evolutionary history [32]–[34]. Given that *Platecarpus* occupies an intermediate phylogenetic position within derived mosasaurs [38] and that its first stratigraphic occurrence is about 10 million years after the earliest record of the family [39], the discovery of LACM 128319 indicates that the advanced aquatic adaptations of mosasaurs (i.e., flipper-shaped limbs, a streamlined body, and demarcated caudal fin functioning as an oscillating foil), as in the other secondarily aquatic tetrapod lineages [30]–[34], are likely to have originated within less than 10 million years after the evolutionary divergence from their terrestrial sister clade (Figure 10).

## Materials and Methods

We directly examined LACM 128319 at the Dinosaur Institute, Natural History Museum of Los Angeles County and photographed the entire specimen using a Nikon D300 camera with an AF-S Nikkor 24–70 mm lens, using softbox lighting and white reflectors. Additional photographs were taken under ultraviolet light using UV-A lamps with a wavelength of 400–320 nm. Measurements were made with calipers. The measurements of vertebral centrum size used herein are condyle (or rear end) height (CH) and centrum length (CL), respectively. In order to document the presence of wedge-shaped vertebral centra, the vertebrae at the tailbend and adjacent caudals were measured along their dorsal and ventral surfaces, respectively.

Samples selected for SEM analysis were mounted on glass slides using double-sided carbon tape and examined using a Hitachi S-3400N scanning electron microscope. Initial screening was performed on uncoated samples under low vacuum, and their elemental composition was determined via EDX analysis. The samples were subsequently sputter-coated with gold to allow better resolution.

A gross dissection of an adult individual of *Varanus exanthematicus* (LO 10298; Department of Earth and Ecosystem Sciences, Lund University) was performed in order to examine the morphology of the respiratory tube, as well as the shape, positions, and relations of various internal organs.

## Supporting Information

Table S1 Measurements of dorsal and ventral centrum length (in mm) at the tailbend, in thirteen caudals that precede the bend and in thirteen caudals that succeed it.
(0.01 MB XLS)
